# The Dialogue Between Two or More Brains: The “Hyperscanning” for Organization

**DOI:** 10.3389/fpsyg.2020.598332

**Published:** 2020-10-30

**Authors:** Michela Balconi, Giulia Fronda

**Affiliations:** ^1^Research Unit in Affective and Social Neuroscience, Department of Psychology, Catholic University of the Sacred Heart, Milan, Italy; ^2^Department of Psychology, Catholic University of the Sacred Heart, Milan, Italy

**Keywords:** hyperscanning, leadership, communication, neuroscience, neuromanagement

## What and Why “Hyperscanning”

When we engage in social exchange, we join information with one another by producing actions and simultaneously adapting to the other person's actions via a sharing of mental representation and action within- and between-individuals (Hari and Kujala, 2009; Konvalinka et al., 2014; Balconi and Vanutelli, 2016; Balconi and Fronda, 2020). It has been shown that this form of adaptation between partners is applied in the case of a common aim and perspective (Masumoto and Inui, 2013; Sacheli et al., 2013; Konvalinka et al., 2014). However, the neural mechanisms underlying this intersubjective coordination of perception and actions remain largely unexplored.

Research in social neuroscience and social science has only recently substituted the study of individual focus and singular brain in isolation, that react to “social” situations, with studies of inter-subjective and dual brains (Sebanz et al., 2006; Konvalinka et al., 2014). This change was suggested by the idea that social minds and brains reactions are intrinsically different when people engage in interaction, rather than act as single individual (Schilbach et al., 2013; Konvalinka et al., 2014).

In this perspective, hyperscanning paradigm can help to support and study this new perspective in neuroscience. It consists in the simultaneous recording of the cerebral activity of two or more subjects involved in social and interpersonal tasks (Balconi and Vanutelli, 2016, 2017b). Therefore, hyperscanning allows exploring interpersonal brain-to-brain responsiveness induced by social interactions. Indeed, previous studies showed that the reciprocal adaptation of two brains in interactions results in a consistent synchrony, as shown by some specific tasks (such as cooperative or competitive tasks) as an example of possible applications of such paradigm.

Indeed, since these tuning mechanisms cannot be measured in isolation (Vanutelli et al., 2015; Balconi and Vanutelli, 2017b), hyperscanning is useful for their investigation in different common interaction contexts, such as communication exchanges or joint-actions.

Specifically, the hyperscanning technique has allowed to abandon the classical experimental paradigms, which focused on the recording of individuals' responses in different social situations (Camerer, 2003). The study of the “social brain,” indeed, is one of the emerging fields in neurosciences that has led, in recent years, to the investigation of the neurophysiological bases of the social behavior of two interacting individuals.

Hyperscanning, indeed, through the use of different neuroscientific tools, such as fNIRS, EEG, functional magnetic resonance imaging (fMRI) and biofeedback, allowed to detect the neural correlates of two or more individuals involved in various social interaction processes (Balconi and Vanutelli, 2016; Balconi et al., 2019a, 2020).

During different social interactions, mechanisms of imitation and joint attention occur between individuals leading to a greater attunement (Frischen et al., 2007). The first application of hyperscanning was concerned with the simultaneously measurement of two individuals' brain activity in different social situations related to the performance of an interactive task (Montague et al., 2002) or during economic exchanges (King-Casas et al., 2005). Other hyperscanning studies have observed the mechanisms of individuals' brain tuning during the performance of real interaction situations, as demonstrated by the study of Lindenberger et al. (2009), who have observed the neural synchronization of some pairs of guitarists.

Similarly, other studies have investigated the mechanisms of neural and peripheral synchronization during the performance of cooperation and competition tasks, the implementation of prosocial behaviors, and during communicative interactions (Balconi and Vanutelli, 2017a; Balconi et al., 2019a, 2020). Specifically, different mechanisms of brain synchronization or de-synchronization were observed during a cooperative situation in which inter-agent individuals had to synchronize their responses during the performance of an attentional task, compare to the competitive condition in which they had to try to answer faster than the partner (Balconi and Vanutelli, 2018). The mechanisms of brain tuning were also observed during a prosocial condition, in which two inter-agent individuals were asked to perform an attentional task before and after a gift exchange (Balconi et al., 2019a). Finally, brain and peripheral synchronization mechanisms were observed during a non-verbal communicative interaction characterized by the use of different gestures aimed at transmitting information affectively, socially, or informatively characterized (Balconi et al., 2020).

In the light of this evidence, hyperscanning, therefore, proves to be an excellent technique for investigating inter-cerebral synchrony mechanisms and communicative and relational coordination (Dumas et al., 2010).

## The Application of Hyperscanning to Leadership Style and Communication

Considering the different leadership styles, such as the transformative and the authoritative one, some recent studies have used the hyperscanning paradigm to investigate fundamental phenomena within the managerial context, such as the moment of employees' evaluation (Balconi et al., 2019b). Indeed, the hyperscanning has shown itself as the more adaptive technique to observe the mechanisms involved in the communicative exchanges between leader and employee. Indeed, the competence to synthonize with the other one involved in the interaction appears to be fundamental for the formation of profitable and productive working relationships (Balconi et al., 2011; Balconi and Canavesio, 2013).

Starting from current literature, which observes how empathy and others' understanding lead over time to brain and body tuning mechanisms, recent research has been interested in investigating the neuro and physiological correlates associated with these tuning mechanisms. Our study also, through the concurrent use of EEG and biofeedback, aimed to recognize the lexical and neurophysiological markers related to the interactions between leader and employees by investigating the neural mechanisms of synthonization associated to the style of leadership, the role played within the organization, and the significant topics revealed by the employee evaluation interview. These points are fascinating because the ability to better interact with others provides some positive outcomes, increasing the sense of self-satisfaction, resilience, and physical and mental well-being, reducing stress levels (Balconi et al., 2019b).

In light of this evidence, the consideration and the analysis of interpersonal, communicative, and emotional processes underlying organizational contexts is essential.

In particular, one of the most examined topic concerns the role of leader and its interpersonal effects on the organizational context. Indeed, efficient relationships between managers and employees can directly cause effects within the organizations (Balconi et al., 2019b). In a recent study, Jiang et al. (2015) have investigated the role of interpersonal neural synchronization (INS) mechanisms in the emergence of leadership, observing if the latter are correlated with communications' quality and frequency.

In particular, to this aim, the cerebral activity of three groups composed of 11 members was recorded with the use of fNIRS in hyperscanning during a leaderless group discussion (LGD) task. The discussion of the groups of participants was video recorded and coded to investigate leadership and communication (Jiang et al., 2015).

From results, a greater level of INS emerged in the left temporo-parietal junction (TPJ), more implicated in processes of social mentalization, within the dyads composed of leader-follower (LF) compared to follower-follower (FF) ones. Specifically, a significant difference has emerged regarding the frequency of communication, which is higher for LF dyads than FF ones. On the contrary, no differences have emerged concerning the frequency of leader initiated and follower-initiated communication (Jiang et al., 2015).

These authors observe that as evidenced by evolutionary theories, which report human and non-human animals' tendency to develop skills useful to compete for survival, human leaders play a fundamental role in maintaining group cohesion (Jiang et al., 2015). Indeed, leaders have to consider their own needs and that of their followers to facilitate the cooperation between the group members.

As interesting demonstrated by different neuroimaging studies, during cooperative situations compared to competitive ones, individuals' neural activity appear to be more synchronized (Cui et al., 2012; Jiang et al., 2015).

Furthermore, the INS is connected to the level of interagents' understanding (Stephens et al., 2010; Jiang et al., 2015), but it is not yet clear whether it is involved in the leader's emergency.

Moreover, it has been shown that high-quality communication increases the level of mentalization of individuals, consisting of the ability to understand social situations by adapting their behavior to others. Verbal and non-verbal communication skills, indeed, have been considered as a fundamental competence of the leader (Jiang et al., 2015). In fact, it is assumed that leaders' emergence occurs in conjunction with the development of good communication skills, relating to knowing how to use the right words at the right time. Through the use of EEG in hyperscanning, an increase in delta activity was observed in leaders assigned a priori to followers, and an increase in INS has emerged from leaders to followers compared to followers to leaders (Sänger et al., 2012, 2013; Jiang et al., 2015).

Also, an increased neural and bodily attunement, demonstrated by the use of gestures, has emerged between leaders and followers assigned a priori during social interactions (Yun et al., 2012; Jiang et al., 2015).

These different applications of hyperscanning within the organizational context open the way to other possible uses of this paradigm for the investigation of different processes and behaviors related to work performance, such as proactive and social resources, useful for implementing more functional strategies for corporate performance (Ingusci et al., 2019; Kim and Beehr, 2020).

Indeed, the increase in individuals' proactive behavior and social relationships could enhance job redesigns' strategies, such as job crafting, with positive effects on workers' well-being (Kim and Beehr, 2020). Furthermore, hyperscanning could prove useful for investigating moral decision-making that appears to have a strong influence on organizational functioning and well-being (Balconi and Fronda, 2019).

## Gesture Synthonization in Functional Communication

Besides observing the mechanisms of brain areas' synchronization and coupling, some studies have shown asymmetric synchronization mechanisms within dyads composed by leader and follower (Dumas et al., 2012; Konvalinka et al., 2014; Balconi et al., 2020). In particular, this asymmetry has been defined, on one side, as functional connectivity, or a partial mechanism of directed coherence between different cerebral regions, as demonstrated by an increase of activation of the prefrontal areas for leader compared to that of ACC/parietal areas of his partner during a card game; on the other side, this asymmetry can be defined as a direct phase of coupling that occur in the activity of frontal alpha frequency band of the brain of leaders to those of the followers (Sänger et al., 2013; Konvalinka et al., 2014). However, no clear evidence has yet emerged on how these phase-connectivity patterns can be configured as a social interaction brain mechanism and how they can be linked to the difference in movement initiation timing.

Our daily social life is characterized by the performance of joint actions, during which we adjust our actions with that of other people according to what is required by different tasks (Zhou et al., 2016). These dynamic adaptations of motor behavior between interagents' individuals during joint actions have been demonstrated by different behavioral studies (Konvalinka et al., 2010; Noy et al., 2011; Zhou et al., 2016). In particular, these adaptation mechanisms are based on a cycle of action and perception (Hari and Kujala, 2009; Zhou et al., 2016) that allows to conduct suitable actions and understand that of others' individuals. Different social cognition studies have paid attention to interacting individuals, thanks to the development of hyperscanning methods using fMRI (Montague et al., 2002; Scholkmann et al., 2013). In particular, Zhou et al. (2016) have analyzed the activity of alpha (7–13 Hz) and beta (13–25 Hz) frequency bands in the sensorimotor cortices, considering their role in the processes of action execution and observation (Hari et al., 1998; Caetano et al., 2007).

In addition, the cerebral mechanisms underlying leadership have been observed by the implementation of a recent fMRI study that required participants a tapping task, including an adaptive stimulus (Fairhurst et al., 2014; Konvalinka et al., 2014). From this study emerged that the management and the perception of leadership appear to be correlated with an increase of activity in right frontal cerebral areas, more involved in self-initiated action.

In particular, this study wanted to implement a two-brain analysis capable of reporting useful characteristics to follow or conduct the behavior of two individuals involved in a realistic dyadic interaction. The use of body movements, indeed, characterizes social exchanges, as happens in gesture communication in which different types of gestures are associated with the transmission of different information (Balconi et al., 2020). In particular, in our recent study, the brain and peripheral tuning mechanisms involved in the observation and reproduction of affective, social and informative positive and negative gestures have been observed during a non-verbal communicative exchange between two subjects. The results of the study revealed an increase in inter-cerebral connectivity in specific brain areas. In particular, regarding hemodynamic activity, an increase in oxygenated hemoglobin (O2Hb) inter-cerebral connectivity in the dorsolateral prefrontal cortex (DLPFC) was observed during the observation of affective gestures and in the superior frontal convolution (SFG) during the reproduction and observation of social gestures. In addition, an increase in O2Hb and inter-brain connectivity in the left side of the DPLFC was observed during the observation of positive gestures. Regarding electrophysiological activity, an increase of inter-brain connectivity for alpha, delta, and theta frequency bands was observed in frontal cerebral regions for affective and social gestures and in posterior cerebral regions for informative ones (Balconi and Fronda, 2020). At autonomic level, an increase of tuning in electrodermal response (level and conductance response, SCL, SCR) has emerged during the observation of negative social and affective gestures.

## Discussion

Therefore, hyperscanning allows exploring interpersonal brain mechanisms generated by social interactions, such as those generally represented in organizations. The mutual adaptation, as shown by brain-to-brain and body-to-body coupling of two or more interactive subjects, results in brain synchrony, which is considered the basic neurophysiological counterpart of functional mutual inter-relationships.

## Author Contributions

MB wrote the first draft and each section of the manuscript. MB and GF contributed to manuscript final writing and revision, read, and approved the submitted version. Both authors contributed to the article and approved the submitted version.

## Conflict of Interest

The authors declare that the research was conducted in the absence of any commercial or financial relationships that could be construed as a potential conflict of interest.
